# Merkel cell polyomavirus and extrapulmonary small cell carcinoma

**DOI:** 10.3892/ol.2013.1483

**Published:** 2013-07-23

**Authors:** KATHRYN C. HOURDEQUIN, JOEL A. LEFFERTS, JEOFFRY B. BRENNICK, MARC S. ERNSTOFF, GREGORY J. TSONGALIS, J. MARC PIPAS

**Affiliations:** 1 Department of Medicine, Section of Hematology/Oncology, Geisel School of Medicine at Dartmouth, Hanover, NH, USA; 2 Department of Pathology, Norris Cotton Cancer Center, Geisel School of Medicine at Dartmouth, Hanover, NH, USA; 3 Dartmouth Hitchcock Medical Center and Norris Cotton Cancer Center, Lebanon, NH, USA

**Keywords:** Merkel cell, polyomavirus, extrapulmonary cancer

## Abstract

The Merkel cell polyomavirus (MCV) is involved in the development of up to 100% of Merkel cell cancer (MCC) cases. Early studies have reported that the virus was infrequently detected in other small cell or neuroendocrine lung carcinomas, which share histological features with MCC. The present study investigated the presence of MCV in cases of extrapulmonary small cell carcinoma (ESCC), which also shares histological features with MCC. A total of 25 cases of ESCC that were diagnosed between 2004 and 2009 were identified at The Dartmouth Hitchcock Medical Center. Archived tissue was available for testing in 16 of these cases. A total of 11 tissue specimens of MCC were used as positive controls. DNA that was extracted from the archived tissue was subjected to five separate quantitative (q)PCR assays for the detection of four MCV genomic targets. MCV DNA was detected in 3/16 (19%) of the ESCCs and in all 11 MCCs. In the three MCV-positive ESCCs, the viral target was only detected by either one or two of the PCR assays. In 8/11 MCV-positive MCCs, the DNA tested positive by either three or all four assays and the remaining three MCCs tested positive by either one or two assays. The β-globin endogenous control was detected in all the samples that were tested. Although MCC and ESCC share numerous histological features, MCV is detected at a lower frequency in ESCC. The possible role for MCV in the etiology of ESCC remains uncertain and may account for the rare cases of ESCC with no other identifiable etiology. The failure of other assays to detect MCV may be due to sequence variability in the MCV genome.

## Introduction

Extrapulmonary small cell cancer (ESCC) is a rare malignancy that arises most frequently in the bladder, prostate, cervix, esophagus, gallbladder, stomach, colon and rectum, larynx, salivary glands and skin. Histologically, the ESCC cells appear similar to those that are present in small cell lung cancer (SCLC), appearing as round to spindle-shaped small cells with dense and inconspicuous nucleoli, sparse cytoplasm and frequent mitoses (1). Immunohistochemically, the tumors stain positive for chromogranin A, synaptophysin and neuron specific enolase (2). ESCC is believed to develop from a pluripotent stem cell that acquires neuroendocrine features (2–4). The cancer acts aggressively, with early and widespread metastases. The incidence is estimated to be 0.1–0.4% (~1,000 cases per year) (3) and the cancer represents 2.5–5% of all cases of small cell carcinoma (4). Overall, the prognosis is poor, with one large series showing three-year disease-free and overall survival rates of 26 and 38%, respectively, and five-year disease-free and overall survival rates of 13% each (5). Studies have reported median survival times of 16.8–43 months in locoregional disease and 2–12 months in patients with distant metastatic disease (6–8).

Merkel cell (neuroendocrine) carcinoma (MCC) of the skin shares numerous features with ESCC. MCCs also exhibit neuroendocrine features and are rare, with ~470 new cases in the United States annually (9). The tumors occur on sun-exposed areas of the head and neck in 29–41% of cases, on the extremities in 40–44% of cases and on the trunk and buttocks in 9–23% of cases (9–11). One-third to one-half of patients develop distant metastases, even if these are not present on the initial presentation. The recurrence rate is 55–79% within the first 6–12 months (10). The one-year survival rates for localized and non-localized disease are 88 and 46%, respectively, with five-year survival rates of 45 and 16%, respectively, compared with expected survival rates in older patients of 92% at one year without MCC and 67% at five years (11).

Histologically, MCC shares numerous features with ESCC, including small blue cells with round to oval hyperchromatic nuclei containing scant cytoplasm, nucleoli that are not prominent and numerous mitoses (12). Immunohistochemically, MCC stains positive for neuron specific enolase, synaptophysin and chromogranin (13). Staining of cytokeratin (CK)-20, a low molecular weight keratin, in a ‘perinuclear dot’ pattern, occurs in 89–100% of Merkel cell tumors and may be used to distinguish MCC from other tumor types. However, 33% of small cell lung cancers (SCLCs) and 3–4% of ESCCs also stain positively for CK20 (14–16). Thyroid transcription factor-1 (TTF-1) is expressed in 83–100% of SCLCs and is absent in MCC (16,17). However, the staining pattern is more variable in ESCCs (42% are TTF-1-positive). Therefore, it is difficult to use this test to distinguish between MCC and ESCC (18).

In 2008, Feng *et al*(19) identified and sequenced a new polyomavirus, the Merkel cell polyomavirus (MCV or MCPyV). MCV sequences were detected in 80% of MCC tumors, but only 8% of control tissues. The observation that DNA was integrated within the tumor genome suggested that MCV infection and integration preceded the clonal expansion of tumor cells and was therefore a possible contributing factor in the pathogenesis of MCC. High levels of MCV DNA in MCC and low levels in other tissues, as well as the likely causative role for MCV in the tumor process, have subsequently been confirmed by multiple other studies (20–25). An improved detection method using novel monoclonal antibodies and new quantitative (q)PCR primers has demonstrated the presence of MCV in up to 100% of MCC specimens (21,22).

SCLC shares histological and immunohistochemical features with MCC, and MCV has been detected in certain SCLCs (24,27). Given the morphological similarities and the similar immunostaining patterns between MCC and ESCC, the present study investigated whether MCV, detectable using PCR, is present in ESCC.

## Materials and methods

### Specimens

This study was approved by the Committee for the Protection of Human Subjects at Dartmouth College and Dartmouth Hitchcock Medical Center (Lebanon, NH, USA). A search of medical records at the Dartmouth Hitchcock Medical Center yielded 25 cases of ESCC that were diagnosed between 2004 and 2009. All the surgical pathology reports were reviewed by a dermatopathologist prior to the analysis of the cases. Archived tissue was available for 16 of these cases. Additionally, 11 tissue specimens from four cases of MCC were used as positive controls.

### DNA extraction and PCR

DNA was extracted from five sections (5 μm each) of the archived formalin-fixed paraffin-embedded (FFPE) tissues. The tissue sections were collected in microcentrifuge tubes, deparaffinzied in xylene, then washed in 100% ethanol, 95% ethanol and phosphate-buffered saline. Genomic DNA was purified from the tissue specimens using Gentra^®^ Puregene^®^ reagents (Qiagen, Valencia, CA, USA), according to the manufacturer’s instructions. Each DNA sample was used in a PCR amplification with four primer sets targeting various regions of the MCV genome and a human β-globin internal housekeeping gene (Table I). qPCR using SYBR-Green detection and melt curve analysis was performed using the ABI 7500 Fast PCR platform (Applied Biosystems, Carlsbad, CA, USA). The reaction mix and running conditions that were used were 12.5 μl 2× SYBR-Green Master mix (Qiagen), 1 μl forward and reverse primer mix (20 μM each), 9.5 μl nuclease-free water and 2 μl DNA (diluted to ~10 ng/μl) at 95ºC for 15 min, followed by 45 cycles of 94ºC for 15 sec, 55ºC for 30 sec and 72ºC for 30 sec. A melt curve analysis was performed using the standard ABI 7500 settings from 60–95ºC at a 1% ramp rate. Quantification cycle (Cq) values were used as quality control indicators.

## Results

MCV DNA was detected in 3/16 (19%) of the ESCC samples and in all 11 MCC samples (from four patients). In the three MCV-positive ESCC cases, the viral target was only detected by either one or two of the PCR assays, while 8/11 MCV-positive MCC DNA samples tested positive by either three or all four assays and the remaining three MCC samples were positive by either one or two assays (Table II). The samples that showed amplification by PCR were confirmed by a melt curve analysis. The β-globin endogenous control was detected in all the samples that were tested.

## Discussion

The present study demonstrated that while MCV was not present in the majority of ESCCs, it was present in several cases, suggesting a role for the virus in rare cases of ESCC. MCV was detected in three of the 16 cases, compared with all 11 of the MCC controls. This suggests that MCV is not as closely associated with ESCC as with MCC, but that it may be a driver of a small number of ESCC cases.

Given the similarities in the histological presentation between MCC and SCLC, Wetzels *et al*(23) investigated the prevalence of MCV in SCLC. MCV was not identified in any of the SCLC tumors. Andres *et al*(24–26) identified a prevalence of 7.5% for MCV in SCLC. However, it was concluded that this reflected the prevalence in the general population, as a similar MCV prevalence was identified in non-MCC tumors of sun-exposed skin and in cutaneous lymphoproliferative disorders. Duncavage *et al*(28) investigated the presence of MCV in SCLC and in other high-grade neuroendocrine tumors, including the gastrointestinal tract, female reproductive system, soft tissue, head and neck region and bladder. MCV was identified in only one of the 74 cases and it was concluded that MCV did not have a role in the oncogenesis of visceral high-grade neuroendocrine tumors. More recently, however, Jung *et al*(22) demonstrated that 37.5% of SCLCs were positive for MCV by PCR. The presence of MCV DNA in a small number of ESCCs in the present study suggests that this virus may play a role in the rare cases of ESCC with unexplained etiology.

The present study targeted four various regions of the MCV genome using a qPCR assay. The MCV-positive ESCC cases demonstrated viral heterogeneity with respect to the regions of the genome that were detected by these assays. This may have been due to the various sets of primers preferentially amplifying sequence variants, due to degraded DNA or due to the amount of viral DNA being below the detection level of the assays. This is supported by the higher quantification cycle (Cq) values in the ESCC samples versus the MCC samples. Therefore, a multitarget approach such as this may be beneficial in detecting MCV. Alternatively, inadequate analytical specificity by certain PCR assays may have resulted in false-positive results due to the presence of viral DNA with sequence similarities to MCV.

Although ESCCs and MCCs have similar morphologies and are stained with similar immunohistochemical markers, they are distinct entities, as evidenced by the difference in CK20 staining between the two groups. ESCC stains diffusely for keratin whereas MCCs have a characteristic juxtanuclear dot-like pattern. Since only one or two of the four assays detected MCV DNA in a limited number of ESCC cases, additional testing, including DNA sequencing, is required to confirm the results in these cases. The failure of the other assays to detect MCV may be due to sequence variability in the MCV genome and may support the presence of an MCV variant in small numbers of ESCC that have not been detectable by routine PCR assays. The present study does not refute the possibility of a role for MCV in the etiology of ESCC, but rather suggests a role for MCV in a small number of ESCCs that may be due to rare MCV sequence variants.

## Figures and Tables

**Table I:** Primer sequences for the various MCV and internal housekeeping targets.

First author, year (ref.)	Target	Forward primer	Reverse primer
Andres *et al,* 2010 (25,26)	MCV_D (2185–2322)	GGTTAGAGATGCTGGAAATGACC	CAAATAAGCAGCAGTACCAGGC
Andres *et al,* 2010 (25,26)	MCV_C (1994–2184)	CCACTTTATTATCTTAGCCCAT	TCCTTTTGGCTAGAACAGTGTC
Wetzels *et al*, 2009 (23)	MCV_A (740–879)	ATCTGCACCTTTTCTAGACTCC	ATATAGGGGCCTCGTCAACC
Duncavage *et al,* 2009 (28)	MCV_B (1072–1179)	TCAGCGTCCCAGGCTTCAGA	TGGTGGTCTCCTCTCTGCTACTG
	β-globin	ACACAACTGTGTTCACTAGC	CAACTTCATCCACGTTCACC

MCV, Merkel cell polyomavirus. Nucleotide positions given are based on NC_010277.1.

**Table II:** qPCR results of the MCC (M1A-M4A) and ESCC (SCC01-SCC25) samples.

Sample	Region A-MCV	Region B-MCV	Region C-MCV	Region D-MCV	β-globin
M1A	Positive	Positive	Positive	Positive	Positive
M1B	Positive	Positive	Positive	Positive	Positive
M1C	Positive	Positive	Negative	Negative	Positive
M1D	Positive	Positive	Positive	Positive	Positive
M1E	Positive	Positive	Positive	Positive	Positive
M2A	Positive	Positive	Negative	Positive	Positive
M2B	Positive	Positive	Positive	Positive	Positive
M3A	Positive	Positive	Negative	Positive	Positive
M3B	Positive	Positive	Negative	Negative	Positive
M3C	Positive	Positive	Negative	Positive	Positive
M4A	Negative	Positive	Negative	Negative	Positive
SCC01	Negative	Negative	Negative	Negative	Positive
SCC02	Negative	Negative	Negative	Negative	Positive
SCC03	Negative	Negative	Negative	Negative	Positive
SCC04	Negative	Negative	Negative	Negative	Positive
SCC05	Negative	Negative	Negative	Negative	Positive
SCC07	Negative	Negative	Negative	Negative	Positive
SCC09	Negative	Negative	Negative	Negative	Positive
SCC10	Negative	Negative	Negative	Negative	Positive
SCC11	Negative	Negative	Negative	Negative	Positive
SCC12	Negative	Negative	Negative	Negative	Positive
SCC15	Negative	Negative	Negative	Negative	Positive
SCC17	Positive	Negative	Negative	Negative	Positive
SCC19	Positive	Positive	Negative	Negative	Positive
SCC21	Negative	Negative	Negative	Negative	Positive
SCC23	Negative	Negative	Negative	Negative	Positive
SCC25	Negative	Negative	Negative	Positive	Positive

Regions A-D are described in the references in Table I. MCC, Merkel cell cancer; ESCC, extrapulmonary small cell carcinoma; MCV, Merkel cell polyomavirus; qPCR, quantitative PCR.
